# The TRPC2 channel forms protein-protein interactions with Homer and RTP in the rat vomeronasal organ

**DOI:** 10.1186/1471-2202-11-61

**Published:** 2010-05-21

**Authors:** Thomas G Mast, Jessica H Brann, Debra A Fadool

**Affiliations:** 1 Department of Biological Science, The Florida State University, Tallahassee, FL, 32306, USA; 2 Program in Neuroscience, The Florida State University, Tallahassee, FL, 32306 USA; 3 Institute of Molecular Biophysics, King Life Science Building, Tallahassee, FL, 32306, USA; 4 Current Address: Columbia University, 914 Fairchild, MC 2439, 1212 Amsterdam Ave., New York, NY 10027

## Abstract

**Background:**

The signal transduction cascade operational in the vomeronasal organ (VNO) of the olfactory system detects odorants important for prey localization, mating, and social recognition. While the protein machinery transducing these external cues has been individually well characterized, little attention has been paid to the role of protein-protein interactions among these molecules. Development of an *in vitro *expression system for the transient receptor potential 2 channel (TRPC2), which establishes the first electrical signal in the pheromone transduction pathway, led to the discovery of two protein partners that couple with the channel in the native VNO.

**Results:**

Homer family proteins were expressed in both male and female adult VNO, particularly Homer 1b/c and Homer 3. In addition to this family of scaffolding proteins, the chaperones receptor transporting protein 1 (RTP1) and receptor expression enhancing protein 1 (REEP1) were also expressed. RTP1 was localized broadly across the VNO sensory epithelium, goblet cells, and the soft palate. Both Homer and RTP1 formed protein-protein interactions with TRPC2 in native reciprocal pull-down assays and RTP1 increased surface expression of TRPC2 in *in vitro *assays. The RTP1-dependent TRPC2 surface expression was paralleled with an increase in ATP-stimulated whole-cell current in an *in vitro *patch-clamp electrophysiological assay.

**Conclusions:**

TRPC2 expression and channel activity is regulated by chaperone- and scaffolding-associated proteins, which could modulate the transduction of chemosignals. The developed *in vitro *expression system, as described here, will be advantageous for detailed investigations into TRPC2 channel activity and cell signalling, for a channel protein that was traditionally difficult to physiologically assess.

## Background

The mammalian accessory olfactory system (AOS) functions as a detector for chemical signals (chemosignals) concerning social organization and conspecific reproductive status [1,2]. The vomeronasal organ (VNO) is the primary sensory organ for the AOS. In rodents, the VNO is an encapsulated neuroepithelium containing a lumen and is able to aspirate fluids via a vascular pump [3,4]. Vomeronasal sensory neurons (VSN) express vomeronasal receptors (VRs) belonging to one of the two families of G-protein coupled receptors (GPCRs) specific to the VNO, the V1Rs and V2Rs [5-7]. Upon binding of a chemosignal, V1Rs and V2Rs activate the G-proteins, Gα_i2 _and Gα_o_, respectively [1]. G-protein activation can ultimately result in a non-specific cation current through the canonical transient receptor potential channel type 2 (TRPC2) [8-10].

VNO function is dependent upon TRPC2 [11-13]. Adaptor proteins scaffold TRPC to proteins such as inositol 1,4,5-trisphosphate receptor type 3 (IP_3_R3) [14-16]. In the invertebrate retina TRP is in a scaffold-mediated complex whereby deletion of the scaffold leads to complex degradation and altered light responses [17]. The TRP channel and IP_3 _receptor are co-localized in VSN microvilli [18] whereby peptide disruption of the protein-protein interaction between them inhibits chemosignal-induced currents [14].

Homers are adaptor proteins that bind to proline-rich sequences on proteins associated with calcium signalling [19]. Long Homer isoforms (1b/c, 2a/b, 3) contain, whereby the immediate-early gene encoded short isoform (1a) lacks, a coiled-coil motif that mediates multimerization [19,20]. Homers alter the function and distribution of metabotropic glutamate receptors (mGluRs) [20-22].

TRPC2 clones have poor surface expression *in vitro *and may require a chaperone for proper formation [23,24]. Receptor transporting protein 1 (RTP1) and receptor expression enhancing protein 1 (REEP1) are putative transmembrane protein chaperones expressed in the main olfactory system, which target olfactory GPCRs to the membrane and form protein-protein interactions with olfactory GPCRs *in vitro *[25]. RTP1 and REEP1 mRNAs are expressed in the mouse VNO, but as of yet, neither protein has been reported in VSNs, and have not been proposed to have any functional interactions with vomeronasal GPCRs [25].

Given the role of TRPC2 in chemosignal detection, the interactions of TRPC2 with adaptor proteins in other sensory systems, and chaperone mRNA expression in the VNO, we sought to test the hypothesis that TRPC2 forms protein-protein associations with partners that could alter channel function or localization. Specifically, we sought to identify interactions between TRPC2 and Homer family members and interactions between TRPC2 and RTP1 or REEP1. First, we demonstrate VNO protein expression of RTP1, Homer, and TRPC2. Next we describe novel, *in vivo*, interactions between TRPC2 and Homer 1b/c as well as TRPC2 and RTP1. Lastly, we demonstrate a physiological role for the interaction between RTP1 and TRPC2; *in vitro *co-expression of RTP1 with TRPC2 leads to increased cell-surface expression of functional TRPC2.

## Results

### Homer, RTP1, and REEP1 are Expressed in the Vomeronasal Organ

**Figure 1:**
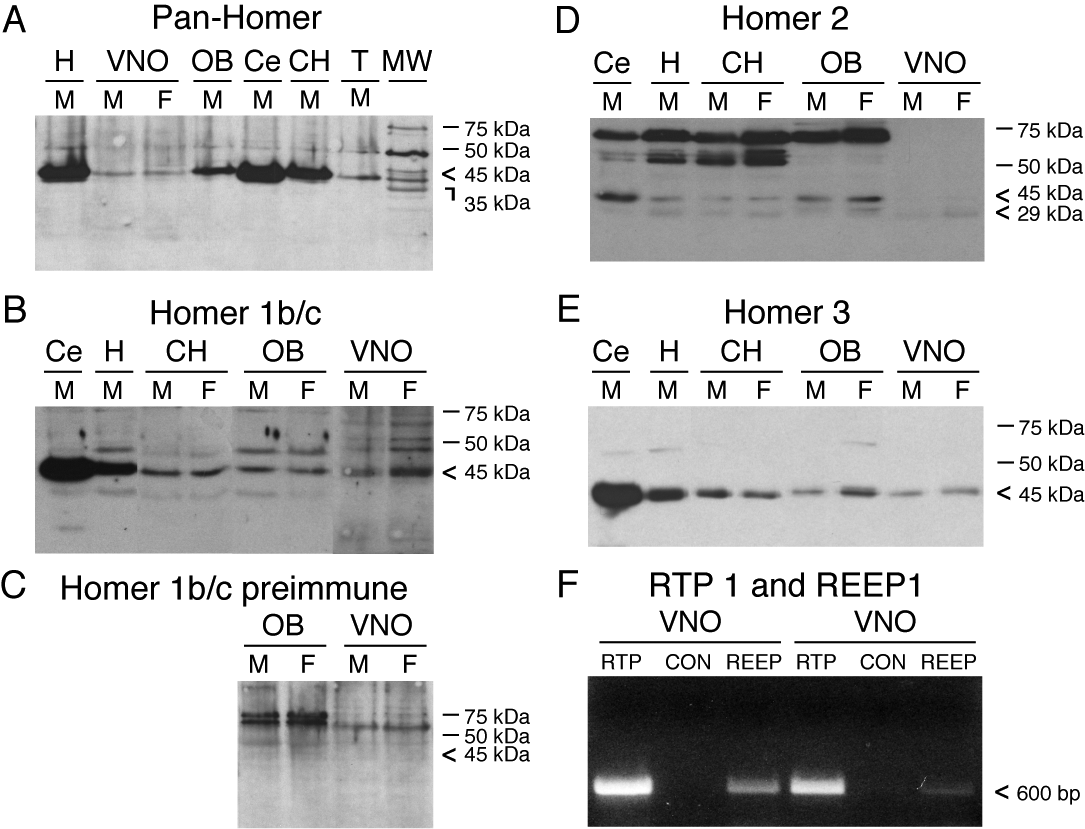
**Adaptor protein expression in the rat vomeronasal organ (VNO)**. (A-E) Representative Western blots of NP40-solubilized proteins incubated with Homer family antisera as labeled. Proteins were separated by SDS-PAGE and then electro-transferred to nitrocellulose as described in the text. Expected Mr = 45/47, 29 kDa. Abbreviations: H = hippocampus, VNO = vomeronasal organ, OB = olfactory bulb, Ce = cerebellum, CH = cerebral hemispheres, T = testis, M = male, F = female. Representative of 3-4 individual preparations for each antiserum. (F) Two representative trials of RT-PCR products specific for RTP1 (RTP) and REEP1 (REEP), respectively. The expected PCR products were 597 bp (RTP1) and 623 bp (REEP1). CON = no reverse transcriptase negative control.

TRPC2 forms a protein-protein interaction with IP_3_R3 in rat VSNs [18] and a peptide blocker of this interaction functionally reduces odor-activated currents [14]. We therefore questioned whether adaptor proteins might moderate the scaffold complex. Rat VNO tissue was screened with an antiserum that recognized all homer isoforms (Pan-Homer). Pan-Homer antiserum was observed to label proteins at the predicted molecular weight via SDS-PAGE followed by Western analysis on NP40-solubilized tissue extracts from the hippocampus (H), cerebellum (Ce), cerebral hemisphere (CH), olfactory bulb (OB), and vomeronasal organ (VNO) (Fig. 1A). Only long forms (M_r _= 45 kDa) were detected using the pan-Homer antiserum, presumably because the short form of Homer (H1a) is a transient product of an activity-dependent immediate early gene [20], making detection difficult. The commercial pan-Homer antiserum (Fig. 1A), and not the investigator-generated antisera (Fig. 1B-E) (see methods), produced a non-selective band at 50 kDa, and therefore was not utilized in subsequent biochemical analyses.

Isoform-specific antisera were then utilized to further probe which Homer isoforms were predominantly found in the VNO. Homer 1b/c and Homer 3 were expressed in all neural tissues tested including the OB and the VNO (Figs. 1B and 1E). Repetition of SDS-PAGE and Western analysis using the same tissue extracts as in Fig. 1B resulted in no immunoreactive labelling with preimmune serum used to generate the homer 1b/c antiserum (Fig. 1C). Homer 2 was weakly detected in the VNO, but was found in the OB, as well as other brain regions tested (Fig. 1D). In our previous studies we have noted sexual dimorphism in the VNO [26,27], therefore, male and female VNO lysates were separately probed with Homer antisera. An appreciable sex difference was not consistently detected (Figs. 1A-E). Equal protein loading was confirmed for all Western blots by stripping the blot and then re-probing for actin immunoreactivity (i.e. Additional File 1, Fig. S1).

RTP1 and REEP1 mRNAs have only been demonstrated in the mouse [25], therefore rat VNO cDNA was screened using gene-specific primers and reverse-transcriptase PCR (RT-PCR) for chaperone expression. RTP1 and REEP1 expression in the rat VNO is reported in Fig. 1F. Each RT-PCR reaction produced a single band and the identity of the band was confirmed by sequencing. An additional screening of RTP1 in the mouse main olfactory epithelium was also performed across various postnatal stages as reported in Additional File 1, Fig. S1.

**Figure 2:**
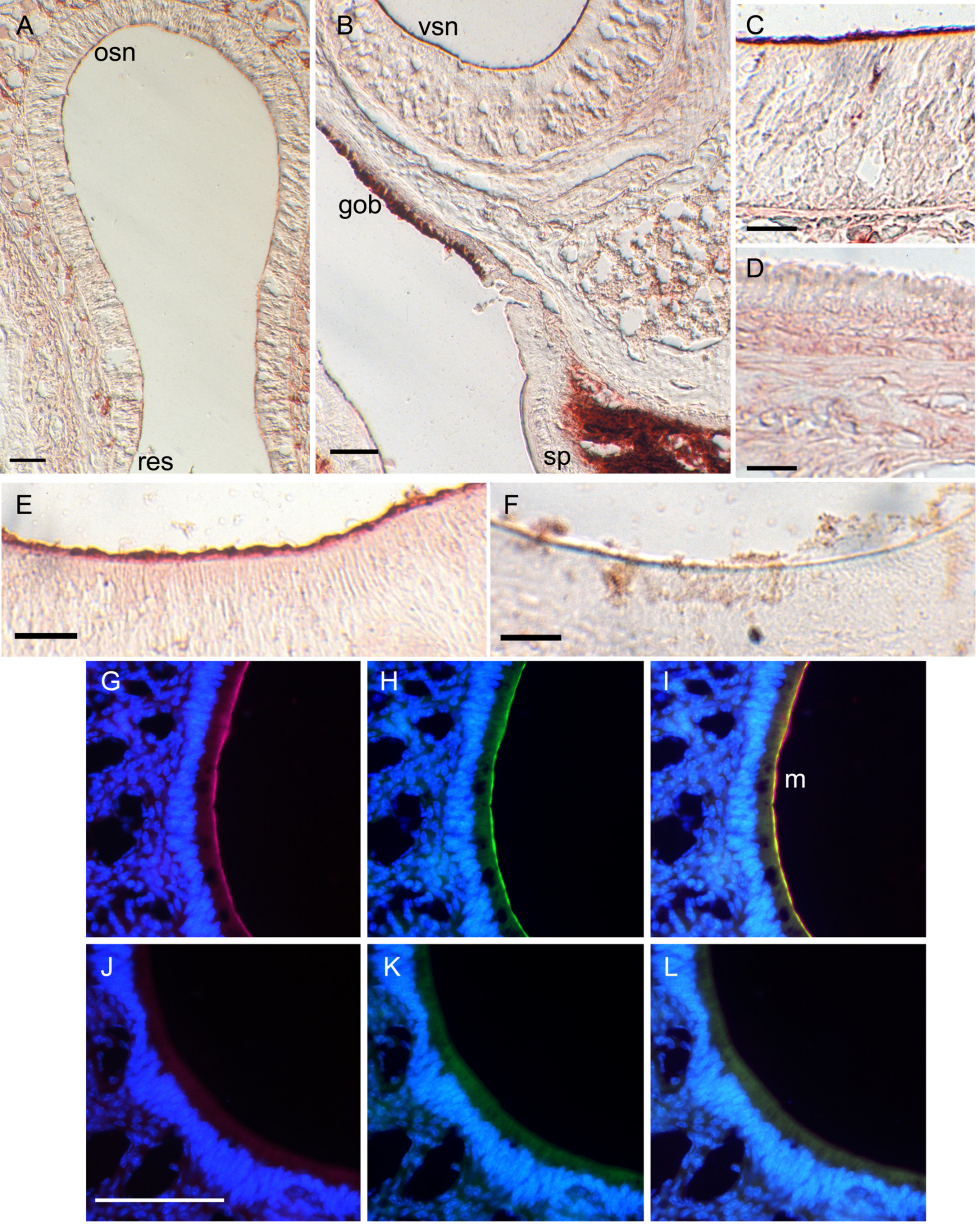
**TRPC2/RTP1 co-localization in the rat nasal cavity**. Sixteen micron coronal sections were processed for αRTP1 immunocytochemistry using an avidin-peroxidase chromagen method (A-F) or dual-colored immunocytochemistry with fluorescent secondary antisera (G-L). Low-power magnification of the (A) main olfactory epithelium (MOE) and (B) vomeronasal epithelium. osn = olfactory sensory neuron, res = respiratory epithelium, vsn = vomeronasal sensory neuron, gob = goblet cells, sp = soft palate. Higher-power magnification reveals αRTP1 labelling in the cilia layer of the MOE (inset C) and microvilli layer of the VNO (inset E) but absence of label in the respiratory cilia (inset D). Control VNO section with omission of primary antiserum (F). Higher-power magnification of the VNO (G-L), αRTP1 (G), αTRPC2 (H), merged image (I). Control sequential sections to that of G-I with omission of the primary antisera (J-L). Scale bar 25 μm (C-F) or 100 μm (A, B, G-L). Scale bar in J is the same for G-L. Italicized lettering in boxes is linked to enlarged image sub-panel. m = microvillar layer.

Since RTP1 cellular localization has not been previously explored in the olfactory system, the determination of the cells or cellular processes that express RTP1 was made using immunoctyochemistry (ICC). Coronal sections of the rat nasal cavity containing both the vomeronasal and the main olfactory epithelium (MOE) were incubated with an antiserum for RTP1 (Fig. 2). Using an avidin-peroxidase chromagen method, putative RTP1 immunoreactivity was evident in the rat MOE cilia (osn; Fig. 2A), VNO microvilli (vsn; Fig. 2B), goblet cells (gob; Fig. 2B), and the soft palate (sp; Fig. 2B). Higher resolution micrographs of the MOE cilia and VNO microvilli can be seen in Figs. 2C and 2E, respectively. RTP1 immunoreactivity was absent from all other structures including the respiratory epithelium (res; Fig. 2D) and from the VNO microvilli that were processed without primary antiserum (Fig. 2F). To determine whether the labelling of RTP1 might overlap with previous TRPC2 microvillar localization reported by our laboratory and others [8,18] we used dual-colored ICC with fluorescently-tagged secondary antisera to test for co-localization of TRPC2 and RTP1. As shown in Figs. 2G-I, the signals for the two proteins extensively overlap at the microvilli (m) as evidenced in the merged image overlay (Fig. 2I). Omission of primary antisera eliminated the fluorescent signal in each condition tested (Figs. 2J-L). As an additional control for RTP1 antiserum specificity, human embryonic kidney 293 (HEK293) cells were transfected with an RTP1 expression vector and then processed under non-permeabilizing conditions for RTP1 immunoreactivity. Pre-incubating the cells with proteinase K abolished the RTP1 immunoreactivity (Additional File 1, Fig. S1B, C).

### Homer and RTP1 form protein-protein interaction with TRPC2

**Figure 3:**
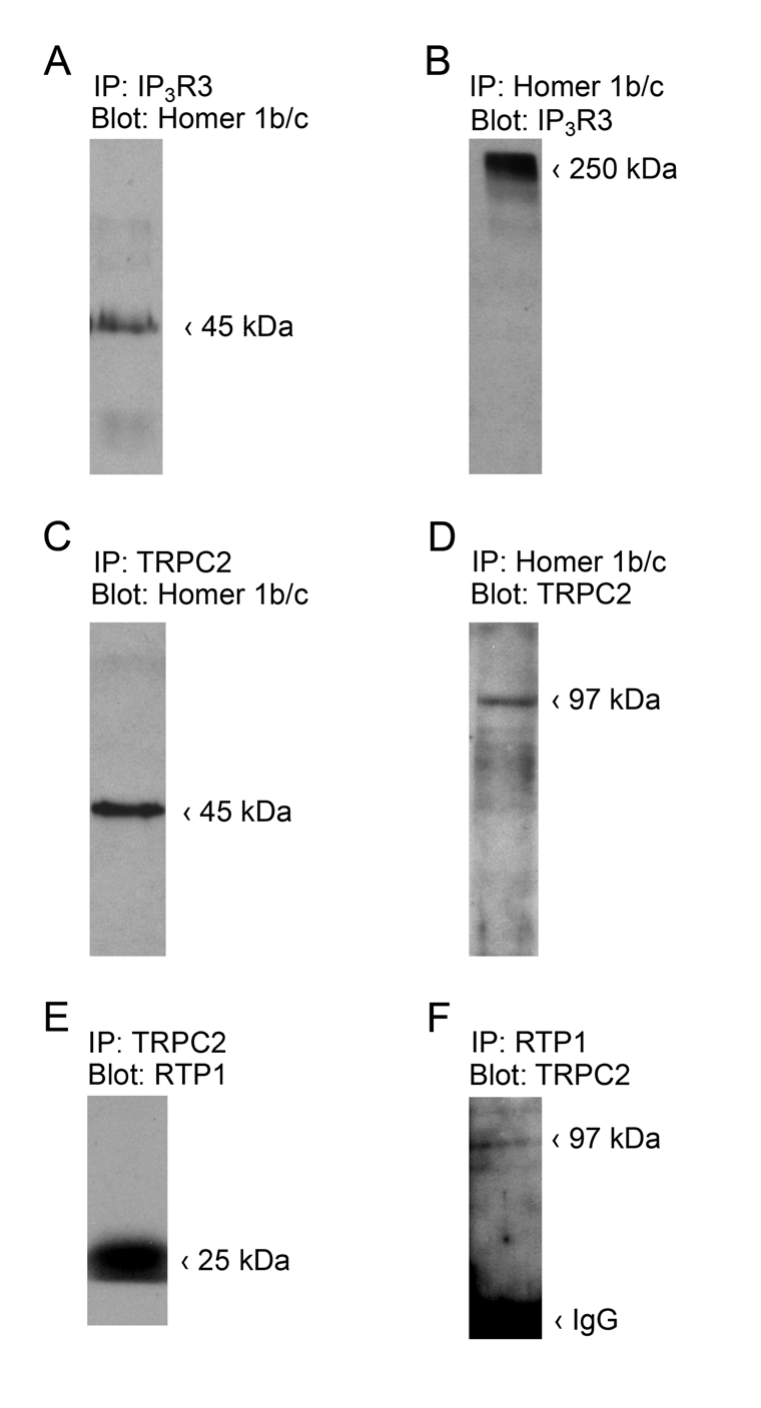
**TRPC2 co-immunoprecipitates with IP_3_R3, Homer 1b/c, and RTP1 in the rat VNO**. For each condition the antiserum used for immunoprecipitation (IP) was added to the VNO lysates, IP proteins were separated by SDS-PAGE and then probed (Blot) with an antiserum against the suspected protein partner. Data are representative for 3-5 individual preparations. Expected Mr = TRPC2 97, IP_3_R3 >220, Homer 1b/c 45 kDa, as indicated. (A) Homer 1b/c co-immunoprecipitated with IP_3_R3. (B) The reverse co-immunoprecipitation of A. (C) Homer 1b/c co-immunoprecipitated with TRPC2. (D) The reverse co-immunoprecipitation of C. (E) RTP1 co-immunoprecipitated with TRPC2. (F) The reverse co-immunoprecipitation of E. IgG = the heavy chain of the immunoglobin G.

Previous *in vitro *data have demonstrated that TRPC2 and Homer 1 form a protein-protein interaction [16]. Our discovered co-localization of TRPC2 and RTP1 suggests that these proteins may also bind *in vivo*. To test the rigor of potential protein-protein interactions, reciprocal co-immunoprecipitations were performed in native rat VNO lysates (Fig. 3). TRPC2 was clearly immunoprecipitated with both Homer 1b/c (Figs. 3C and 3D) and RTP1 (Figs. 3E and 3F). Interestingly, Homer 1b/c could co-immunoprecipitate with both IP_3_R3 (Fig. 3A and 3B) and TRPC2. Additional negative controls using rabbit sera as the first source for immunoglobulins failed to produce bands at the expected molecular weight (Additional File 1, Fig. S1D). Protein interactions with REEP1 could not be investigated due a lack of available antiserum.

### TRPC2 transfection efficiency in a heterologous expression system

**Figure 4:**
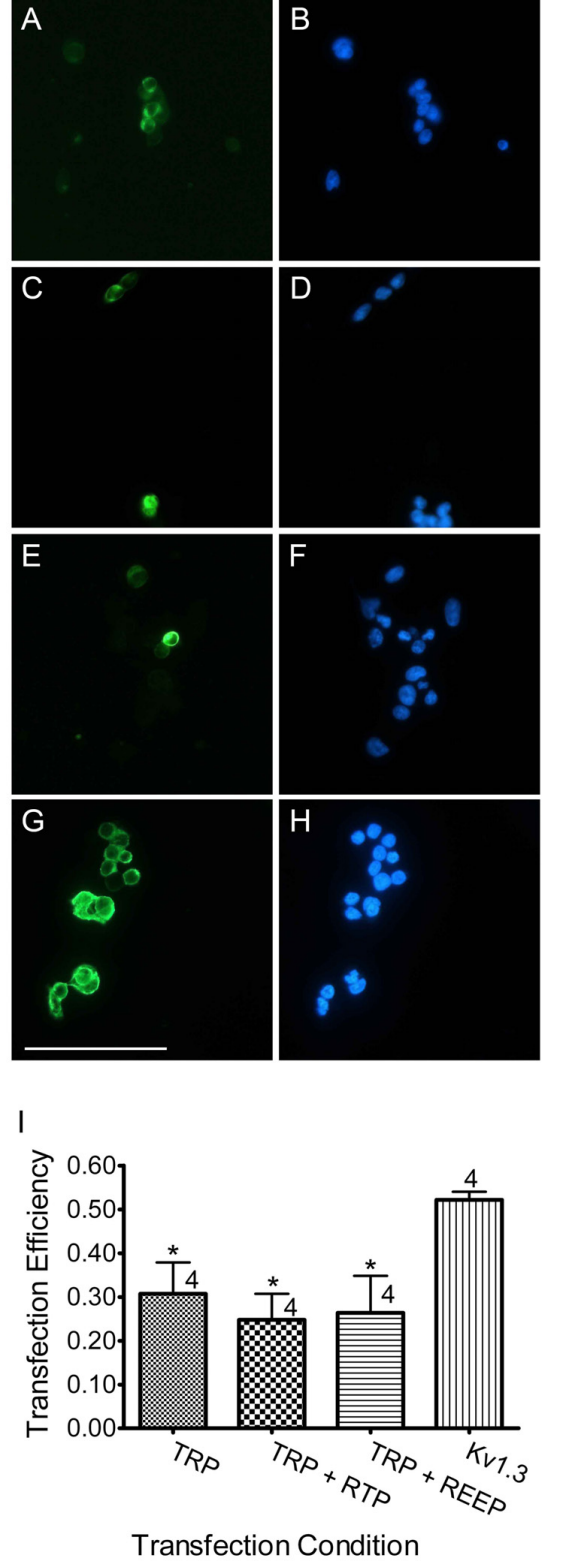
**TRPC2 transfection efficiency in HEK293 cells**. (A-H) Representative micrographs of cells grown on glass-coverslips, transfected as in text, incubated with an antiserum to the c-myc epitope and labeled with FITC-conjugated secondary antiserum. (A, C, E, and G) Representative fields of view for antiserum specific fluorescence (green). (B, D, F, and H) The same fields of view as in images B, D, F, and H with DAPI nuclear stain (blue). Note the low percentage of cells transfected in (A, B) TRPC2, (C, D) TRPC2 + RTP1, (E, F) TRPC2 + REEP1, as compared to (G, H) Kv1.3. The scale bar in G is 100 μm. (I) Histogram plot of the ratio of transfected cells. The number of quantified transfections are noted. * = denotes a mean pixel density significantly different from Kv1.3 transfection; one-way ANOVA followed by a *snk *post-hoc test (p ≤ 0.05).

Due to the reported increased surface expression of olfactory receptors by the chaperone RTP1 [25] and our discovery of its interaction and cellular co-localization with TRPC2, we hypothesized that the chaperone would increase the surface expression of TRPC2. To test this, TRPC2 was expressed with or without either RTP1 or REEP1 in HEK293 cells. Transfected cells were incubated with an antiserum for the myc-epitope tag on the plasmid-encoded TRPC2 and then visualized using a species-specific FITC-conjugated secondary antiserum. A representative field of view used to calculate the transfection efficiency for various transfection conditions is shown in Fig. 4. The ratio of fluorescent cells (left column, Figs. 4A, C, E, and 4G) over that of all cells (right column, Figs. 4B, D, F, and 4H; DAPI nuclear stain) was used to calculate transfection efficiency for each condition, respectively (Fig. 4I). TRPC2 transfection by itself (Figs. 4A and 4B) and in conjunction with either RTP1 (Figs. 4C and 4D) or REEP1 (Figs. 4E and 4F) resulted in an efficiency near 30% (Fig. 4I). Transfection with another myc-epitope tagged six transmembrane spanning ion channel (Kv1.3) was used as a positive control (Figs. 4G-H). The TRPC2 transfection condition efficiencies were significantly below that of the positive control as measured by a one-way ANOVA followed by a *Student Newman-Keuls *(*snk*) post-hoc test (F = 4.01, p < 0.05). Neither cells transfected with empty vector nor those labelled without the primary antiserum were immunoreactive (data not shown).

### RTP1 alters the subcellular localization of TRPC2 *in vitro*

**Figure 5:**
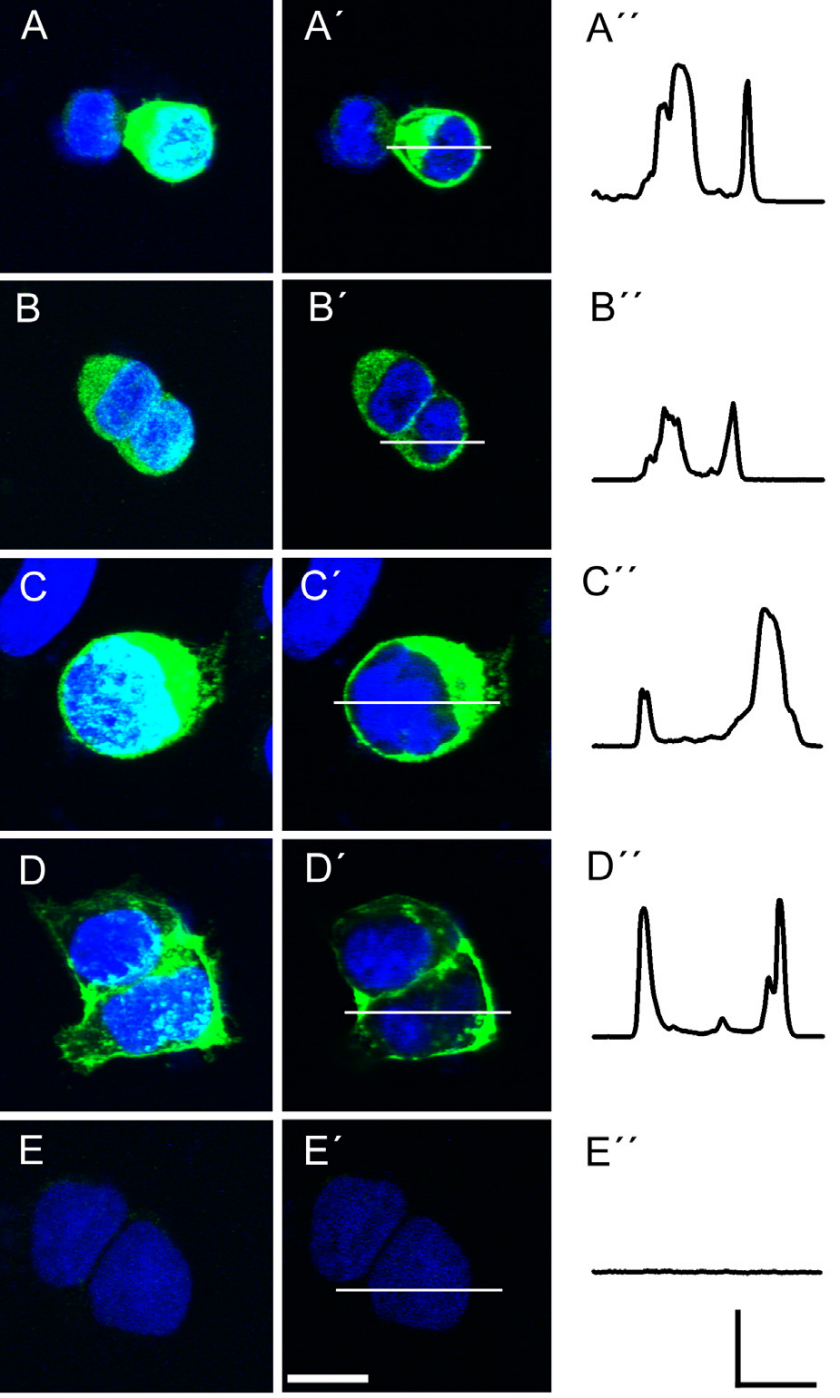
**Subcellular localization of TRPC2 in HEK293 cells**. (A-E) Representative two-photon confocal micrographs of cells grown, transfected, and immunolabeled as in Fig. 4. Note that TRPC2 immunoreactivity in (A) appears subcellular while that of Kv1.3 immunoreactivity (D) is localized to or compartmentalized near the membrane. The presence of RTP1 (B) or REEP1 (B-C) appears to shift some TRPC2 immunoreactivity towards the membrane while that in the presence of REEP1 (C) remains more cytosolic or intermediate. Immunoreactivity is abolished in a vector-only transfection in E (pcDNA_3_). Horizontal scale bar = 10 μm. Vertical scale bar = 50 arbitrary units. (A-E) = Z-series stacked confocal image; (A'-E') = Representative single optical slice in the midline; (A''-E'') = Intensity profiles quantified as a line scan taken at the horizontal bar in A'-E'.

RTP1 and REEP1 are able to induce membrane expression of olfactory receptors in HEK293 [25]. Therefore, we sought to visualize the effect of these chaperones on the subcellular distribution of TRPC2 in the HEK293 cells. The identical transfection scheme as in Fig. 4 was repeated but high-resolution images of the labelled cells were acquired using confocal microscopy. The results of three such experiments are shown in Fig. 5. When expressed alone, TRPC2 immunoreactivity is visualized either near the nucleus (light blue; Fig. 5A) or in dense patches seemingly away from the surface membrane (bright green) and more easily viewed in an unstacked image (Fig. 5A'). In contrast, Kv1.3 immunoreactivity is visualized primarily at the surface membrane (Fig. 5D, 5D') and is known to insert into the surface membrane in large numbers in HEK293 cells. Interestingly, co-expression with RTP1 (Fig. 5B, 5B') results in immunoreactivity that is more typical of surface expression, with less immunoreactivity near the nucleus (ie less light blue), than that observed for the TRPC2 alone condition. Co-transfection with REEP1 results in an intermediate distribution (Fig. 5C, 5C'). Transfection of HEK293 cells with TRPC2 and both chaperones did not appear different than that of cells using transfection conditions with only one chaperone in conjunction with TRPC2 (data not shown). Cells labelled without the primary antiserum were not immunoreactive (Fig. 5E, 5E'). Line plots of each transfection condition in Fig. 5A-E demonstrate the α-myc immunoreactivity distribution.

**Figure 6:**
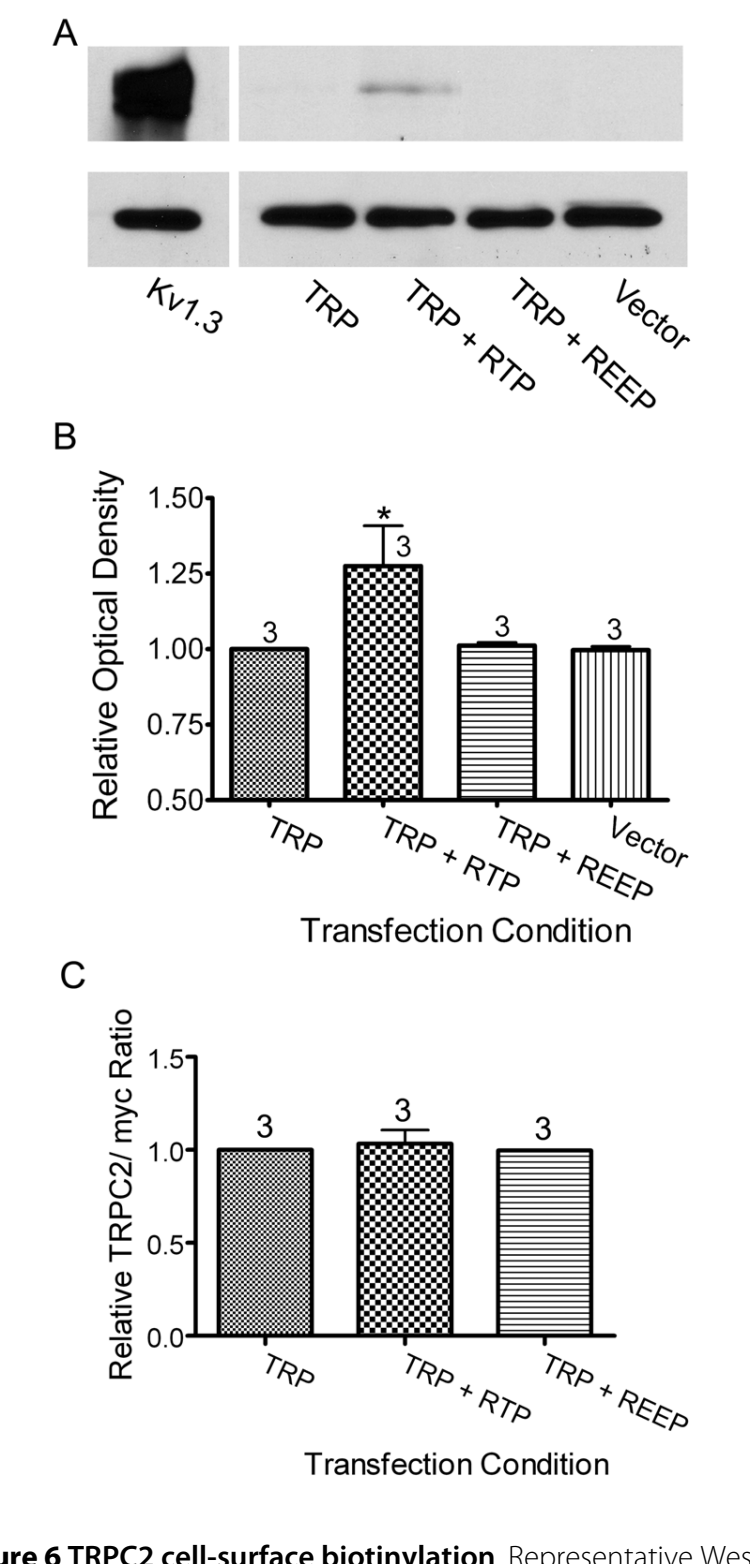
**TRPC2 cell-surface biotinylation**. Representative Western blot of TRPC2-biotinylated protein following 48 hours after transfection of indicated cDNAs in HEK293s. (A) (Top panel) Biotinylated protein products (see methods) were precipitated with streptavidin agarose beads, subjected to SDS-PAGE, and then probed with α-c-myc (1:400) to visualize myc-Kv1.3 expression (far left lane) versus that of mycTRPC2 expression under various transfection conditions. (Bottom panel) Lysate input probed with α-β-actin. (B) Histogram plot of the mean ± s.e.m. pixel immunodensity normalized to that of the TRP alone condition. Transfection sample size noted. *= Mean pixel density is statistically-different from channel only transfection; Dunnett's post-hoc test, p ≤ 0.05. (C) Ratio of the expression levels of mycTRPC2 and the internal standard, myc protein. Values are normalized against the channel only condition.

To biochemically confirm the suggested increase in surface expression of TRPC2 by RTP1 and REEP1, a set of cell-surface biotinylation experiments were conducted on HEK293 cells transfected as in Fig. 5. The biotinylated surface proteins were collected and processed by SDS-PAGE and visualized via Western analysis using an antiserum to the myc-epitope. Cell lysates were also processed by SDS-PAGE and visualized via Western analysis for β-actin to confirm equivalent protein loading. A representative Western blot of biotinylated TRPC2 channel is reported in Fig. 6 as quantified 48 hrs following transfection in register with subsequent functional physiological experiments below. RTP1, but not REEP1, significantly increased TRPC2 surface biotinylation (Significantly-different mean pixel density; one-way ANOVA followed by a Dunnett's post-hoc test F = 4.1, p < 0.05; Fig. 6B). When transfected alone, surface TRPC2 was detected in only 2 out of 7 trials; while when transfected with RTP1, surface TRPC2 was detected in 7 out of 7 trials. Comparatively, myc-tagged Kv1.3 was strongly detected under either condition (Fig. 6A).

### Cell-surface TRPC2 is functionally detected

**Figure 7:**
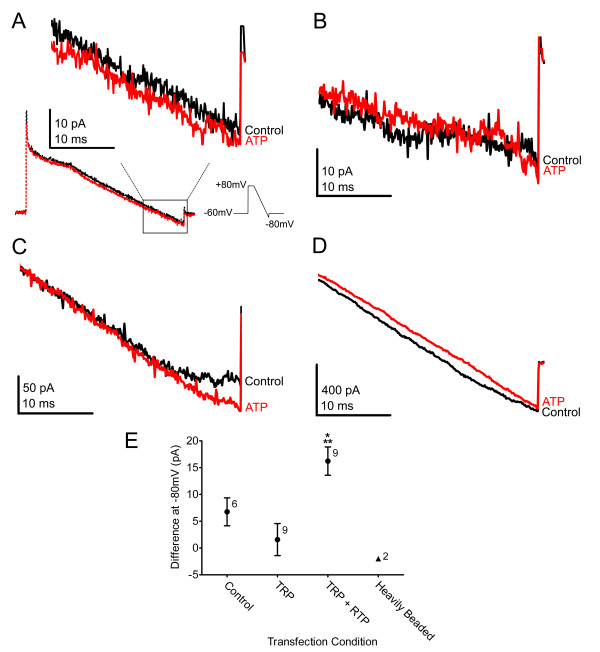
**Whole-cell electrophysiology**. (A-D) Human embryonic kidney 293 (HEK293) cells were transiently transfected with cDNAs encoding the TRPC2 channel, hCD8 and with or without RTP1 as noted. HEK293 cells were voltage-clamped at -60 mV in the whole-cell configuration and then a ramp was applied (+80 to -80 mV; see methods) under control (black) and subsequent to ATP bath application (red). (A) Representative control (beadless) whole-cell recording. The bottom recordings are of the entire voltage protocol. The box outlines the portions of these recordings shown in greater detail in the inset above the dashed lines and used in C-D. Representative recordings from the TRPC2 alone (B), TRPC2+ RTP1+ (C), and the heavily beaded TRPC2+ RTP1+ (D) conditions. (E) Graph of the change in mean (± s.e.m) current at -80 mV following ATP stimulation as compared to a previous stimulation under various transfection conditions as in (A-C). Solid black triangle = denotes the mean response to ATP stimulation by heavily beaded cells as in (D). Transfection sample size as noted. * = denotes mean current is statistically different from control transfection; ** = denotes mean current is statistically different from TRPC2 transfection; one-way ANOVA followed by a *snk *post-hoc test (p ≤ 0.05). The voltage-ramp protocol is described graphically in (A).

To confirm that the cell-surface expressed TRPC2 was functional, a set of whole-cell electrophysiological experiments were conducted on HEK293 cells transfected as in Fig. 5. Dynabead technology using co-transfection of the channel with CD8 was employed to pan for transfected cells appropriate for whole-cell patch-clamp (see methods for details). Transfected HEK293 cells that were lightly beaded (2-4 beads) and apparently exiting mitosis were targeted in order to facilitate the generation of consistent, comparable recordings across several transfections. Cells were stimulated with a voltage-ramp protocol as described in [9] and graphically displayed in Fig. 7A. The total duration of the ramp was 140 ms with an interpulse interval of 60 s. Three to five ramp pulses were applied to determine baseline current and then the recording bath was changed (see methods) to yield a final ATP concentration of 166 μM. Although diacylglycerol (DAG) has been reported to activate endogenous TRPC2 channels in mouse VSNs [9], in our hands neither DAG nor a synthetic analogue, OAG, was effective in gating TRPC2 current in vertebrate VSNs [14]. We therefore relied upon reported augmentation of TRPC2 via activation of purinergic current [24] as the metric of a functional assay of our *in vitro *system. Current at the end of the voltage-ramp protocol (-80 mV) was used as a measure of response to ATP stimulation. As shown in Fig. 7C, cells transfected with both TRPC2 and RTP1 elicited an increase in ATP-evoked whole-cell current compared to that of control cells that were transfected, but beadless (Fig. 7A) or TRPC2-transfected only cells (Fig. 7B) (Significantly-different mean current; one-way ANOVA followed by a *snk *post-hoc test F = 7.68, p < 0.05; Fig. 7E). Additionally, a different population of heavily-beaded cells (N = 7) were observed, which had much larger voltage-activated current amplitudes that ranged from 400 to 4400 pA (mean = 1550 pA). These cells were minimally responsive to ATP (Fig. 7D, E) and were only observed in dishes transfected with both TRPC2 and RTP1.

## Discussion

These experiments demonstrate that the putative olfactory receptor chaperone RTP1 interacts with not only proteins of the GPCR superfamily, but also with ion channels. This novel finding was demonstrated *in vivo *by a protein-protein interaction between TRPC2 and RTP1, and *in vitro *by a RTP1-dependent increase in TRPC2 surface expression. An ion channel complex consisting of TRPC2, Homer, and IP_3_R3 may exist *in vivo*. Our biochemical experiments indicate that TRPC2, the scaffold protein Homer 1b/c, and the ion channel IP_3_R3 form protein-protein interactions in the native VNO. To the best of our knowledge, an interaction between Homer and TRPC2 has not been demonstrated in any sensory system. Lastly, our data represent the first characterization of Homer expression in the rat VNO.

**Figure 8:**
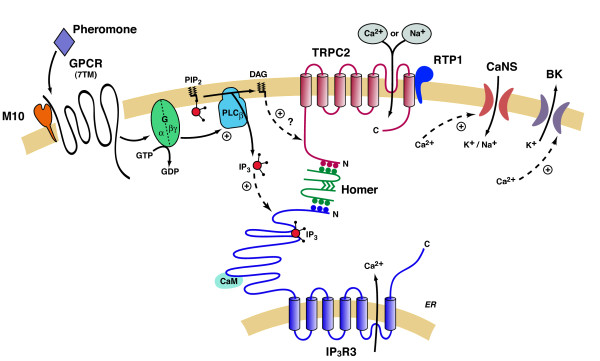
**Schematic of the hypothetical vomeronasal organ transduction model**. The vomeronasal organ (VNO) signal transduction pathway begins at either a type 1 vomeronasal (V1R) or type 2 vomeronasal (V2R) G-protein coupled receptor (GPCR); V2Rs may be associated with a member of the M10 major histocompatibility protein family. Upon pheromone binding, the V1R/V2R activates a guanidine trisphosphate-binding protein (G-protein). The activated G-protein stimulates the cleavage of phosphatidylinositol 4,5-bisphosphate (PIP_2_) into 1,4,5-inositol trisphosphate (IP_3_) and diacylglycerol (DAG) via phospholipase C (PLC). DAG has been reported to gate the non-specific cation current through the type 2 canonical transient receptor potential channel (TRPC2). Although binding of IP_3 _to the IP_3 _receptor (IP_3_R) is not supported to yield a transduction current alone, two isoforms of IP_3_R (IP_3_R2 and IP_3_R3) are expressed in the VNO, and IP_3_R3 forms a complex with TRPC2. Whether or not ligand occupancy is required, inhibition of complex formation blocks chemosignal-activated current. TRPC2 expression is increased in the VSN microvillar membrane with the assistance of the chaperone receptor transporting protein 1 (RTP1), whereby the channel associates with both Homer 1b/c and IP_3_R3. A calcium activated non-selective ion channel (CaNS) can amplify the chemosignal-induced current following the rise in intracellular calcium. This rise in intracellular calcium also actives calcium-activated big conductance potassium ion channels (BK) as well as calmodulin (CaM), which may bind to both IP_3_R3 and TRPC2.

Homers are widely expressed in both the male and female rat including, but not limited to, many brain regions and the testes. Although these biochemical data cannot exclusively rule out the possibility of Homer expression in non-sensory regions, Homers are typically associated with either post-synaptic membranes [20,22,28,21,31]or membranes involved in calcium signalling [19]. The VNO lysates used in these experiments contained both of these subcellular elements, consistent with previous expression patterns for Homer. The non-sensory portions of the VNO are densely innervated by the autonomic nervous system [3,32] and the sensory microvilli contain a well-explored calcium-signalling pathway (Fig. 8) [1,14,18]. Homer isoforms expressed in the VNO could function similarly in smooth muscle cells and in chemosensory signal transduction. Unlike mouse data reported by [33], we do not find Homer 2 expression in the rat VNO. Overall, we detected neither a sex difference in VNO expression of Homer 1b/c and 3 isforms nor an appreciable amount of the Homer 2 isoform.

Our data demonstrating transcription of RTP1 and REEP1 in the rat VNO support the previous finding of these transcripts in the mouse VNO [25]. The fact that our immunocytochemical characterization supports the detection of RTP1 protein in olfactory sensory neuron cilia, VSN microvilli, goblet cells and in the soft-palate while RTP1 is not detected in the non-sensory respiratory epithelium, may provide important clues as to function. *In vitro *experiments support RTP1 interactions with GPCRs associated with either odorant [25] or taste receptor families [34]. Similar protein-protein interactions have not been found, however, with VRs [25].

Alternatively, MHC class 1b proteins have been found to associate with the V2Rs and thus may not function with TRPC2 [35,36]. Allowing for several different protein associations including M10-VR, RTP1-TRPC2, and Homer-TRPC2-IP_3_R3 provides the pheromone transduction cascade with multiple regulatory sites (Fig. 8).

The immunoprecipitation data indicate that Homer is not expressed in the non-sensory areas of the VNO, rather it is expressed in VSN microvilli. Other data have indicated that TRPC channels can interact with Homer proteins [16]. For example, TRP (the *drosophila *homologue of TRPC [37]) is involved in such interactions in the invertebrate photoreceptor [17]. Functionally, Homer expression in the VSN would allow for receptor and channel modulation. Linking TRPC2 and IP_3_R3 via Homer 1 would ensure high-fidelity transmission of the calcium signal that flows through the open TRPC2 channel during chemosignal detection. Homer 3 does not form protein-protein interactions with TRPC2 [16]. The inducible form of Homer (1a isoform) was not detected in the rat VNO. This short form lacks the coiled-coiled domain and would oppositely be predicted to disassemble a TRPC2-Homer 1-IP_3_R3 complex (see Fig. 8). Disruption of this interaction could alter TRPC2 activity in a manner similar to Homer 1a modulation of mGluR activity [21]. In previous VSN recordings, disruption of the interaction between TRPC2 and IP_3_R3 resulted in a diminished chemosignal response [14]. On the other hand, TRPC1 mutants lacking Homer binding sites formed spontaneously active channels when expressed *in vitro *[16] and gene-targeted deletion of Homer 1 increased TRPC1 activity *in vivo *[16,38]. Thus, Homer 1 could provide the VSN signalling apparatus with flexibility in responding to and adapting to chemosignals. The interaction between TRPC2 and RTP1 could provide further regulation of TRPC2 and the response of VSNs to chemosignals, by modulation of total TRPC2 activity dependent upon surface expression driven by the chaperone. The recognition sequence for RTP1 binding will need to be investigated in future experiments.

The plasmid containing TRPC2-C14 had a low transfection efficiency in HEK293 cells. Transient transfection of a plasmid containing Kv1.3 produces an efficiency percentage of approximately 60% [39], whereas TRPC2 efficiency is only about 30%. Poor transfection efficiencies have been noted of some constructs using polycationic transfection reagents [40]; however, other researchers have tried alternative transient transfection methods with TRPC2 and reported comparably low efficiencies [23,41]. It is interesting to speculate that the low transfection efficiency might be a result of apoptosis due to calcium cytotoxicity associated with spontaneously active TRPC2 channels. At least one other TRPC channel, TRPC4, has been demonstrated to be spontaneously active in HEK293 cells [42]. In light of the facts that neither chaperone increased TRPC2 transfection efficiency nor did this efficiency match that of Kv1.3, a structurally similar channel [43], suggests that proper TRPC2 cellular distribution and function may require binding partners not present in our experiments. Lastly, RTP1 and REEP1 do not appear to be toxic to the HEK293 cells, as transfection efficiency did not decrease with their expression.

RTP1 and REEP1 appear to alter the sub-cellular distribution of TRPC2 *in vitro*. In HEK293 cells, TRPC2 immunolabeling is predominately in large deposits that are presumably vesicles, as imaged with laser confocal microscopy. Although every transfection condition with TRPC2 led to vesicular immunolabeling, the presence of either RTP1 or REEP1 seemed to shift expression of the channel toward the surface membrane. These data indicate a functional relationship for the RTP1-TRPC2 co-immunoprecipitation found in VNO tissue. Our data cannot distinguish the mechanism of TRPC2 surface expression, which could be the result of either increased TRPC2 inserted or inhibition of TRPC2 internalization.

Demonstration of robust Kv1.3 surface expression lends credence to the assumptions made earlier when comparing sub-cellular localization of TRPC2 in different transfection conditions. Namely, when transfected with chaperone, the TRPC2 immunolabeling signal was similar to the Kv1.3 immunolabeling in terms of subcellular distribution. A similar shift in the immunolabeling signal of olfactory receptors occurs when these receptors are expressed *in vitro *with RTP or REEP [25] and with unrelated GPCRs [44]. It then follows that with the addition of chaperone, more TRPC2 is in the surface membrane. In support of this notion, in each transfection condition where TRPC2 and RTP1 were transfected together, TRPC2 was detected in the surface membrane. When expressed alone, TRPC2 was infrequently detected in the surface membrane. Increased TRPC2 surface expression was detected with both cell-surface biotinylation and with whole-cell electrophysiology.

The endogenous metabotropic ATP receptor pathway utilized in our experiments to investigate *in vitro *TRPC2 current is similar to the VNO sensory transduction pathway as each activates PLC (Fig. 8) [24,45,46]. The electrophysiological data indicate that the surface expressed TRPC2 is functional and able to respond to a signal transduction pathway similar to that present in the VNO. That heavily-beaded, and presumably highly expressing, TRPC2+ RTP1+ cells were minimally responsive to purinergic stimulation is not unexpected. TRPC3, another TRPC channel, gains and loses agonist-induced activity based on expression level [47]. Alternatively, the TRPC2 protein level may have been high enough and the purinergic receptor protein level low enough, that any interaction between the two might be negligible due to stoichiometric limitations.

Regardless of the mechanism, these data suggest that the half-life residence of TRPC2 in the membrane is increased in the presence of RTP1. These results may indicate that the functional interaction between RTP1 and TRPC2 is one of membrane stabilization rather than trafficking. This is a different functional role for RTP1 from previously described [25] and is speculative. With both olfactory and gustatory GPCRs, RTP1 is presumed to traffic its target to the surface membrane [25,34].

Based upon our current results in the context of current knowledge [1,48,49], we propose the following model (Fig. 8). A chemosignal binds to either VR-type GPCR, activation of which ultimately results in a TRPC2-dependent calcium influx across the surface membrane. Calcium can also enter the cytosol from the endoplasmic reticulum via IP_3_R3. Homer 1 binding at either TRPC2 terminus [50] (residues 303PPTLL and 953LPVPF) may alter the channel function. TRPC2 and mGluRs are both integral transmembrane proteins and, therefore, the known interactions of mGluR/IP_3_R [28] could be replaced by TRPC2/IP_3_R complex formation in the VNO. For example, Homer 1 may cluster IP_3_R3 to TRPC2 using Homer binding motifs (PPXF, PPXXF and LPSSP) on both channels in a similar fashion that Homers cluster IP_3_R3 to mGluR [51]. This would be advantageous for at least two reasons. First, the products of phospholipase C hydrolysis of PIP_2_, IP_3 _and DAG, gate both IP_3_R3 and TRPC2. Maintaining close proximity of the second-messenger targets would increase the speed of the signalling cascade and decrease the amount of second-messenger lost due to errant diffusion. Second, as IP_3_R3 may adopt a conformation that favors opening upon calcium binding [52], tethering IP_3_R3 next to the calcium source would increase the speed of the signalling cascade. By tethering IP_3_R3 underneath TRPC2, Homer would be expected to increase the open probability of IP_3_R3 using calcium influx contributed through the surface channel TRPC2. Thus, Homer could both increase the speed and the efficiency of the TRPC2 signalling cascade as it does for mGluR cascades. Signal adaptation is likely to come from calcium-calmodulin inhibition of both IP_3_R3 [53] and TRPC2 [54], calcium-activated big conductance potassium channels (BK) [55,56] and metabolism of DAG into the lipid arachidonic acid [56,57].

Although complexes of channels and adaptors have been found in the visual system, Homer is a relatively newly discovered protein, and appears to be involved in scaffolding, targeting, and localization. Our previous finding of a direct protein-protein interaction between IP_3_R3 and TRPC2 in the VNO neither ruled out an additional role for scaffolding proteins, such as the Homer family, nor a role for chaperones, such as REEP1 and RTP1. In fact, the addition of chaperones and the formation of an adaptor complex may be critical to channel function and eagerly warrants future experimentation.

## Conclusions

The VNO expresses members of the Homer protein family. TRPC2 complexes with both IP_3_R3 and Homer1b/c *in vivo *in the VNO. RTP1 is expressed in the VNO, co-localizes with other members of the VNO transduction pathway and may be a member of the transduction pathway as it forms a protein-protein interaction with TRPC2 *in vivo*. *In vitro*, RTP1 appears to function as a chaperone of TRPC2, increasing the amount of functional channel in the surface membrane.

## Methods

### Animal care and maintenance

Postnatal Day 30 (P30) Sprague-Dawley rats were used for biochemistry experiments and were housed on a 12 h:12 h light:dark cycle in the Florida State University (FSU) vivarium. All procedures were performed in accordance with the FSU Animal Care and Use Committee and NIH-approved guidelines.

### Solutions

Solutions used for protein sample or tissue preparation, including phosphate buffered saline (PBS), lysis buffer (LB), wash buffer (WB), and protease and phosphatase inhibitor solution (PPI), were made as described in [58]. Tissue extract buffer (TEB) was prepared as in [22]. Cell-surface biotinylation solutions, including biotinylation lysis buffer and biotinylation quench buffer, were made as described by [59]. Immunoblot stripping buffers, including tris stripping buffer (TSB) and sodium citrate stripping buffer (SCSB), were also made as described in Colley *et. al*. (2007). Electrophysiology solutions were prepared as in [24] and were as follows (in mM): intracellular pipette 150 KCl, 10 HEPES pH 7.2, 2 MgCl_2_, 10 glucose; extracellular bath 140 NaCl_2_, 10 HEPES pH 7.4, 4 KCl, 4 CaCl_2_, 1 MgCl_2_, and 10 glucose. All chemicals were obtained from either Sigma Chemical Company (St. Louis, MO, USA) or Fisher Scientific (Suwanne, GA, USA).

### Plasmids and antibodies

All encoded cDNAs were downstream from a cytomegalovirus (CMV) promoter. TRPC2 clone 14, with an N-terminal myc-epitope (EQKLISEEDL), was prepared in the pcDNA_3 _vector and was a kind gift from Dr. L Birnbaumer (National Institute of Environmental Health Sciences) [10]. RTP1 and REEP1 were in the pCI vector and were kind gifts of Dr. H. Matsunami (Duke University) [25]. pCDM8 was a kind gift from Dr. Brian Seed (Harvard University) [60]. DNA encoding human CD8 was amplified from pCDM8 and subcloned into the pcDNA_3 _vector (Carlsbad, CA, Invitrogen) between the BamH1 and EcoR1 restriction sites. cDNA encoding Kv1.3 was subcloned into the pcDNA_3 _vector (Carlsbad, CA, Invitrogen) at the unique HindIII restriction site within the multiple cloning region [39]. Kv1.3 was also epitope-tagged via insertion of the myc sequence on the extracellular face of the channel between the S1 and S2 transmembrane domains [58].

T1NH, T2NH, and T3NH are anti-peptide polyclonal antibodies specific for the type-1, -2 and -3 IP_3_R isoforms, respectively, and were raised against the following amino terminal sequences (amino acid position in parentheses): T1NH = CLATGHYLAAEVDPDQEVDPDQ-DASR (308-326), T2NH = CPDYRDAQNEGKTVRDGKTVRDGELP (320-338) and T3NH = CENPSYKGDVSDPGDVSDPKAAGPGA (319-337). These antibodies were a generous gift of Dr Gregory Mignery (Loyola University Chicago, Stritch College of Medicine, Maywood, IL, USA) [61]. An antiserum detecting TRPC2 as raised in guinea pig and directed against the N-terminal cytosolic domain CSSDASGAGPGGPLRNVE was a generous gift of Dr. Richard Axel (Columbia University, New York, NY, USA) [12]. Anti-peptide polyclonal antibodies detecting the different forms of Homer were made by immunizing rabbits with the synthetic C-terminal peptides of Homer 1b/c (IFELTELRDNLAKLLECS), 2a/b (GKIDDLHDFRRGLSKLGTDN), or 3 (RLFELSELRE-GLARLAEAA) conjugated to thyroglobulin with glutaraldehyde [22]. A fourth, rabbit polyclonal antiserum recognizing all Homer 1 (1a, 1b, and 1c) isoforms was generated against the full-length GST-Homer 1a fusion protein [20]. These antibodies were a generous gift of Dr. Paul Worley (Johns Hopkins University, Baltimore, MD, USA). A polyclonal antibody recognizing all Homer proteins (Pan-Homer; 1a, 1b, 1c, 2a, 2b, 3) was made by immunizing rats with recombinant Homer 1a (AB5875, Chemicon/Millipore, St. Louis, MO, USA). The mouse monoclonal antiserum for the c-myc epitope was from Roche (Indianapolis, IN, USA). Rabbit polyclonal antiserum specific to RTP1 was a kind gift of Dr. Hiro Matsunami (Duke University, Durham, NC, USA) [62]. When necessary to validate equal protein loading, a rabbit polyclonal antibody detecting cellular actin was used. The immunogen for this antibody was SGPSIVHRKCF attached to a Multiple Antigen Peptide (MAP) backbone (Sigma Chemical).

### Tissue homogenization and Western blotting

Tissue extracts were prepared as in [22]. Briefly, P30 rats were killed by CO_2 _inhalation, decapitated, and VNOs were rapidly dissected and placed in ice cold TEB with PPI. Samples on ice were sonicated (Sonic Dismembrator, Model 60, Fisher Scientific) twice for eight seconds on setting number five. Non-soluble matter was removed by ultracentrifugation (Beckman Coulter, Fullerton, CA, USA) at 37,000 × *g *for 30 min at 4°C. The supernatant was aliquoted and stored at -80°C until use. The resultant pellet was solubilized in 2% sodium dodecyl sulfate (SDS), aliquoted, and stored at -80°C in case the initial screening of the lysates did not yield membrane-associated fractions.

Immunoprecipitations were performed as in [18]. In brief, P30 rat VNOs were homogenized with a size 20 Kontes glass tissue-homogenizer (Kontes Glass, Vineland, NJ, USA) on ice in LB with PPI. Lysis was continued on a Roto-Torque (Model 7637, Cole-Parmer Instruments, Vernon Hills, IL, USA) for 30 min at 4°C. Lysates were clarified by centrifugation at 15,000 × g (Eppendorf, Model 5415C, Westbury, NY, USA) for 10 min at 4°C and then precleared for 1 hour (hr) with 3 mg/ml protein A sepharose (GE Healthcare, Uppsalla). This was followed by another centrifugation step to remove the protein A sepharose. Proteins of interest (Homer 1b/c, Homer 2, Homer 3, TRPC2, IP_3_R3, RTP1) were immunoprecipitated from the clarified lysates by overnight incubation on a Roto-Torque at 4°C with 5 μg/ml of appropriate antiserum. Samples were then incubated for 3 hr with protein A sepharose and centrifuged as above. Immunoprecipitates were washed four times with 5 volumes of WB. Lysates and washed immunoprecipitates were diluted in SDS gel loading buffer containing [63] 1 mM Na_3_VO_4 _and stored at -20°C until use.

Purified VNO tissue extracts or immunoprecipitated proteins were separated on 6 to 15% acrylamide gels by SDS-PAGE and electro-transferred to nitrocellulose membranes. Equal protein loading (30 μg) was controlled by Bradford protein assay (BioRad, Hercules, CA, USA) and confirmed by 0.1% Fast Green staining and α-β-actin labeling. The nitrocellulose membrane was blocked with 5% non-fat milk for 60 min, incubated overnight at 4°C in primary antisera against TRPC2 (1:2000), IP_3_R_3 _(1:2000), RTP1 (1:1000), Homer 1b/c (1:1000), Homer 2a/b (1:2000), Homer 3 (1:2000), Homer 1 isoforms (Worley, 1:1000), all Homer isoforms (Chemicon, 1:1000), or RTP1 (1:1000). Membranes were then incubated with horseradish peroxidase-conjugated species-specific secondary antibody (donkey anti-rabbit, Amersham Biosciences or rabbit anti-guinea pig, Sigma) for 90 min at room temperature (rt). Labelled protein was detected with enhanced chemiluminescence (ECL; Amersham Biosciences, Piscataway, NJ, USA) using Classic Blue autoradiography film BX (MidSci, St. Louis, MO, USA). To ensure equal loading, nitrocellulose membranes were stripped by incubating blots in eight ten-minute washes of TSB, followed by eight ten-minute washes of SCSB, then re-probed using α-β-actin (1:1000). Autoradiographs were scanned using a Hewlett-Packard Photosmart Scanner (Model 106-816; Hewlett-Packard, San Diego, CA, USA) and quantified by line scanning densitometry using Quantiscan Software (Biosoft, Cambridge, UK).

### RNA extraction and reverse-transcriptase PCR

P30 rats were killed by CO_2 _inhalation, decapitated, and VNOs were rapidly dissected from the surrounding tissues and placed on dry ice. RNA was extracted using the SV Total RNA Isolation System as per the manufacturer's protocol (Promega, Madison, WI, USA). RNA purity and concentration was determined by UV spectroscopy (NanoDrop-1000, Wilmington, DE, USA). cDNA was reverse-transcribed using the ImPromII kit as per manufacturer's protocol (Promega). Gene-specific primers have been previously reported [34] and were as follows: RTP1_forward, AAGCGTGACCACAGATGAGTG; RTP1_reverse, GAGCAGAAGTTCCAGCCTGAG; REEP1_forward, CAATGAATTCCCACCATGGTGTCATGGATCATCTCCAGGC; REEP1_reverse, GACTAGCGGCCGCCTAGGCGGTGCCTGAGCTGCTAG. PCR products were resolved using 1.0% agarose gel electrophoresis and visualized via UV excitation of the incorporated ethidium bromide.

### Human embryonic kidney cell (HEK293) maintenance and transfection

HEK 293 cells were maintained in minimum essential medium (MEM), 2% penicillin/streptomycin, and 10% FBS (Gibco BRL). Before transfection, cells were grown to confluence (~7 days), dissociated with trypsin, and re-plated to low density onto Corning substrate-coated plasticware on poly-d-lysine-treated glass coverslips as previously described [39]. HEK293 cells were transfected for 4-5 hrs at 37°C with plasmid cDNA and LipofectamineTM transfection reagent (Invitrogen, Carlsbad, CA, USA) in OptiMEM serum-reduced media (Gibco BRL). Either 1.5 μg of plasmid DNA and 7.5 μl Lipofectamine were applied to 30-50% confluent glass coverslips in 35 mm dishes or 3.0 μg of plasmid DNA and 15 μl Lipofectamine were applied to 80-95% confluent 60 mm dishes, for immunocytochemical and biochemical experiments, respectively. pcDNA_3 _vector was used to normalize total DNA concentration in co-transfection conditions, as previously [64].

### Immunocytochemistry

Thirty-six hrs post-transfection, HEK293 cells were washed in PBS and fixed in ice-cold St. Marie's fixative (1.0% acetic acid in 95% ethanol). Cells were washed three times in PBS and incubated for 30 min at rt in PBST-block (0.1% Triton x-100 in PBS and 1% Bovine Serum Albumin). Cells were immunolabeled with primary antisera diluted in PBST-block for 90 min at rt or overnight at 4°C with α-c-myc. Cells were then washed three times in PBS. The secondary antisera were applied at rt for 1.5 hr in PBS using fluorescein isothiocyanate-conjugated goat anti-mouse antiserum (1:200). Following three washes in PBS, cells were counter-stained with a five-minute incubation in diamidino-phenyindole (DAPI) in PBS (1:5000). The cells were mounted on glass slides with Vectashield (Vector Laboratories, Burlingame, CA, USA) to prevent photobleaching.

Tissue sections (~16 μm) were prepared from P30 rats that had been fixed-perfused (4% paraformaldehyde) and sucrose cryoprotected as previously described [18]. Sections were air-dried on the bench for 60 minutes, re-hydrated with PBS, and then incubated at 80°C in 10 mM sodium citrate for 30 minutes for antigen retrieval [65]. The sections were cooled and non-specific binding was blocked by a 60-minute incubation in blocking solution (5% normal goat serum/2.5% BSA/0.3% triton in PBS). During the block, the primary antibodies were treated with L-glutathione to cap reactive SH-functional groups. On ice, an L-glutathione/Tris-EDTA solution was diluted in the blocking solution containing the primary antiserum to a final concentration of 30 mM L-glutathione Tris-EDTA/1.25% normal goat serum/0.625% BSA/0.075% triton [66]. After the block, sections were rinsed with Tris-EDTA - 0.3% triton and incubated with antiserum overnight in a darkened, humidified chamber. Antiserum for RTP1 was used at a final dilution of 1:200 and antisera for TRPC2 was used at 1:400. Sections to be stained using the ABC method were treated according to the manufacturer's protocol (Vector, Burlingham, CA, USA) and the precipitate was visualized with the chromagen AEC (Sigma). Immunofluorescence was detected with either donkey anti-rabbit Texas Red (Amersham Biosciences) or rabbit anti-guinea pig FITC (Sigma).

### Microscopy

Conventional light microscopy was performed on an Axiovert S-100 (Zeiss, Thornwood, NY, USA) equipped with epifluoresence, an AxioCam camera (#412-312, Zeiss), and Axiovision data capture software (version 3.1, Zeiss). Images were captured with a pixel resolution of 1300 × 1030. Laser confocal microscopy was performed on an Axioplan 2 microscope attached to an LSM510 two-confocal system (Zeiss). FITC was excited at 488 and DAPI at 700 nm with argon/2 and titanium/sapphire lasers, respectively. Images were captured at 1024 × 1024 pixels resolution in LSM file format, and then were converted to a 16-bit TIFF file format using LsMB software (Zeiss). The TIFF file was opened in NIH ImageJ http://rsb.info.nih.gov/ij/ and a uniform rectangle of 200 pixels × 20 pixels was applied across the center of the cell (Z-axis) to obtain the plot density profile of the pixels underneath, as previously described [67].

### Transfection efficiency analysis

Three to nine fields of view for each transfection condition (TRPC2 + pcDNA_3_, TRPC2 + RTP1, TRPC2 + REEP1, and Kv1.3 + pcDNA_3_) were captured under a fluorescent and either a brightfield or a DAPI emission. Each field of view was approximately 90,000 μm^2^. Transfection efficiency was calculated for each field of view as the ratio of the number of fluorescent cells divided by the total number of cells. Cell counts were performed using NIH ImageJ software.

### Cell-surface biotinylation

Cell-surface biotinylation was performed as described [59]. Briefly, either 24- or 48-hr post-transfection, HEK293 cells were washed with ice-cold PBS and then incubated with 1.0 mg/ml biotin (Pierce, Rockford, Ill, USA) in PBS for 30 min at 4°C. Following a PBS rinse, cells were incubated for 30 min at 4°C in quench buffer, rinsed again, and then lysed for 30 min at 4°C in lysis buffer. Lysed cells were scraped, triturated, and centrifuged at 12,000 rpm for 10 min at 4°C. The supernatants were collected and aliquots were set aside for confluency controls. Protein concentration was calculated using a Bradford assay (BioRad). Equal amounts of protein were brought up to 1 ml with PBS (pH 8.0) and incubated overnight at 4°C with 40 μl streptavidin-conjugated agarose-beads (Pierce). The beads were pelleted with a centrifugation of 12,000 rpm for 10 min at 4°C, washed, and then stored at -20°C until further use.

### Whole-cell electrophysiology

Hoffman modulation contrast optics was used to visualize cells at 40× magnification (Axiovert 135, Carl Zeiss). Thirty-six hrs post-transfection, HEK293 cells were rinsed with bath solution and incubated with anti-hCD8 beads (Dyna-Beads, Invitrogen) to mark transfected cells (either hCD8+/TRPC2+/RTP1- or cCD8+/TRPC2+/RTP1+) [60]. Co-expression with CD8 allows visualization of cells taking up the cDNA encoding the channel or receptor of interest by marking transfected cells with a red polypropylene-antibody-linked bead. We [64] and others [58] have demonstrated that single cells take up multiple constructs equivalently and that the density of the beads is proportional to the expression of channel of interest. Cells were rinsed two times before beginning a recording session to remove any unbound beads. Cells were rinsed two more times before beginning a recording session. Electrophysiological records were analyzed using software from Microcal Origin (Northampton, MA) and Quattro Pro (Borland International, Scotts Valley, CA).

Patch electrodes were fabricated from Jencons glass (Jencons Limited, Bridgeville, PA) with pipette resistances between 9 and 14 MΩ. Macroscopic currents were recorded in the whole-cell configuration using an Axopatch-200B amplifier (MDS Analytical Technologies/Axon Instruments, Sunnyvale, CA), filtered at 5 kHz, digitized at 5 kHz, and stored for later analysis. All voltage signals were generated and data were acquired with the use of an Axon Digidata 1200 board with pClamp v9.2 software (Axon Instruments). Cells were held routinely at a holding potential (V_h_) of -60 mV, stepped to 80 mV (V_c_), held for 40 milliseconds (ms), and then changed to a ramp protocol by falling to -80 mV over 100 ms (-1.6 mV/ms). The total pulse duration was 140 ms and the inter pulse interval was 60 seconds. Several sweeps were taken over the 3-6 minutes after establishing the whole-cell configuration to establish a stable baseline. ATP stimulation was achieved with bath application of 0.5 ml of 500 μM for a final bath concentration of ~166 mM ATP. Peak inward current amplitude was measured at -80 mV from a subsequent sweep.

### Data analysis

Numerical data were statistically analyzed using Prizm software (version 4, GraphPad, San Diego, CA, USA). One-way Analysis of Variance (ANOVA) with either a Student-Newman-Keuls (*snk*) or a Dunnett's post-hoc test was performed with statistical significance determined at the 95% confidence interval.

## Authors' contributions

TGM carried out the experiments employing immunocytochemistry, electrophysiology, biotinylation, and RT-PCR. Both TGM and JHB conducted the immunoprecipitation and SDS-PAGE/Western experiments. DAF directed the performed research. All authors conceived the experiments, evaluated the design of controls, and wrote sections of the manuscript. All authors read and approved the final manuscript.

## Supplementary Material

Additional file 1 **Fig. S1 - RTP1 Antiserum Characterization and Immunoprecipitation Controls**. (A) NP40-solubilized samples of mouse main olfactory epithelia (MOE), collected at noted postnatal (P) stages, were separated by SDS-PAGE and electro-transferred to nitrocellulose. RTP1 antiserum recognized the appropriate band (expected Mr = 25 kDa). The faint band at the arrowhead may be an RTP1 variant described previously in the MOE [62], but not observed in the VNO (Fig. 3). The top blot probed for the RTP1 chaperone (RTP1) was stripped and reprobed for β-actin (Actin)(Mr = 42 kDa). (B-C) HEK293 cells transfected with the RTP1 expression vector were incubated at 37° with either (B) PBS or (C) 200 μg/μl proteinase K. Cells were immunolabelled with αRTP1 without detergent and processed for RTP1 immunoreactivity as described previously [68]. Note the loss of RTP1 immunoreactivity in (C). (D) VNO lysates were used in immunoprecipitation experiments as in Fig. 3, except non-immune rabbit sera was used as the source of the first immunoglobulin. Note the loss of bands at the expected M_r _for TRPC2 97, IP_3_R3 >220, Homer 1b/c 45, and RTP1 25 kDa as indicated. IgG = the heavy chain of the immunoglobin G. Click here for file
