# Supplementary figures and images for: The TRPC2 channel forms protein-protein interactions with Homer and RTP in the rat vomeronasal organ
Source: BMC Neurosci. 2010 May 21;11:61. doi: 10.1186/1471-2202-11-61 (PMC2881103; doi:10.1186/1471-2202-11-61)

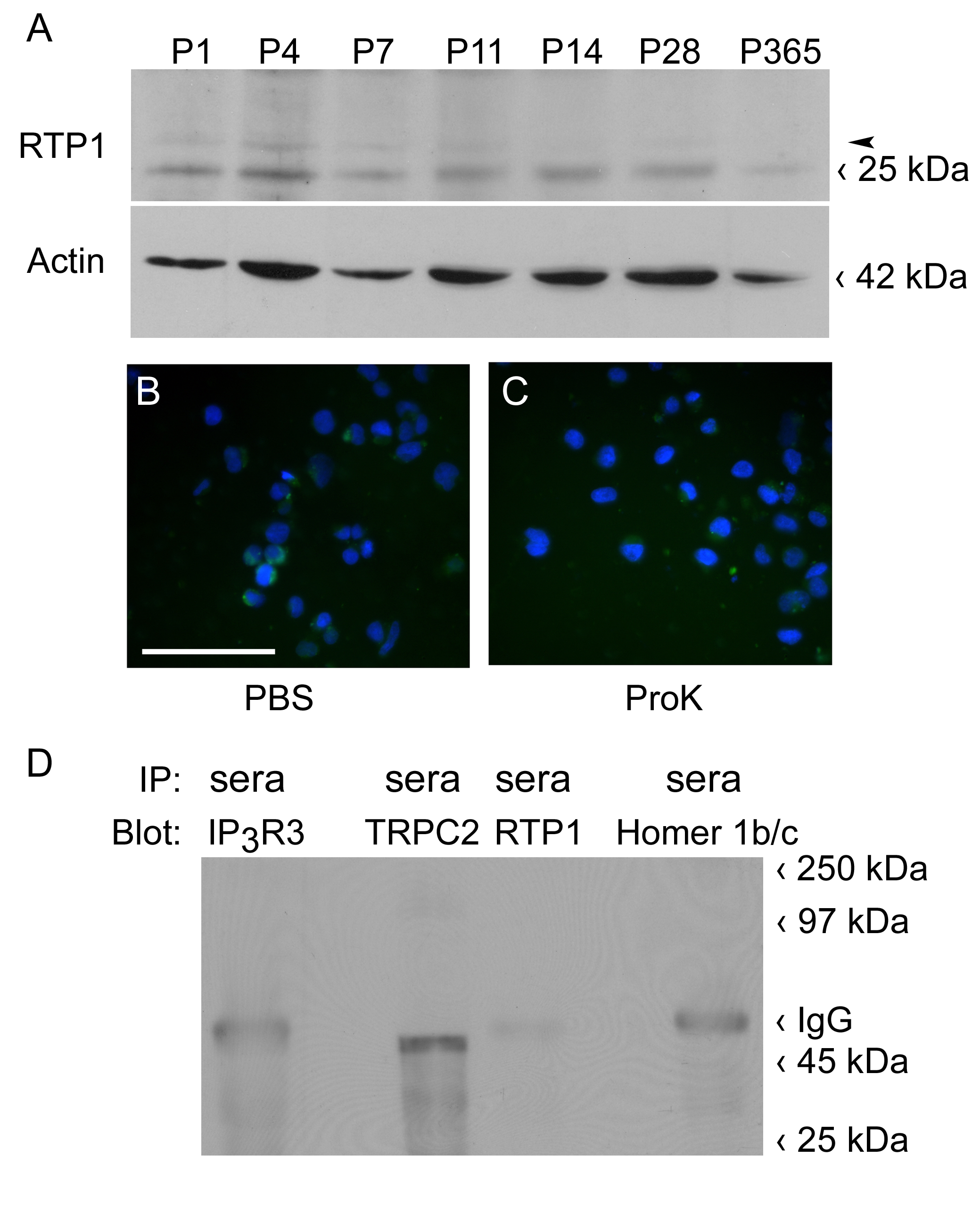

Supplement: Additional file 1 — Fig. S1 - RTP1 Antiserum Characterization and Immunoprecipitation Controls. (A) NP40-solubilized samples of mouse main olfactory epithelia (MOE), collected at noted postnatal (P) stages, were separated by SDS-PAGE and electro-transferred to nitrocellulose. RTP1 antiserum recognized the appropriate band (expected Mr = 25 kDa). The faint band at the arrowhead may be an RTP1 variant described previously in the MOE [62], but not observed in the VNO (Fig. 3). The top blot probed for the RTP1 chaperone (RTP1) was stripped and reprobed for β-actin (Actin)(Mr = 42 kDa). (B-C) HEK293 cells transfected with the RTP1 expression vector were incubated at 37° with either (B) PBS or (C) 200 μg/μl proteinase K. Cells were immunolabelled with αRTP1 without detergent and processed for RTP1 immunoreactivity as described previously [68]. Note the loss of RTP1 immunoreactivity in (C). (D) VNO lysates were used in immunoprecipitation experiments as in Fig. 3, except non-immune rabbit sera was used as the source of the first immunoglobulin. Note the loss of bands at the expected Mr for TRPC2 97, IP3R3 >220, Homer 1b/c 45, and RTP1 25 kDa as indicated. IgG = the heavy chain of the immunoglobin G. [file 1471-2202-11-61-S1.TIFF]
